# Acoustic and Linguistic Features of Impromptu Speech and Their Association With Anxiety: Validation Study

**DOI:** 10.2196/36828

**Published:** 2022-07-08

**Authors:** Bazen Gashaw Teferra, Sophie Borwein, Danielle D DeSouza, William Simpson, Ludovic Rheault, Jonathan Rose

**Affiliations:** 1 The Edward S Rogers Sr Department of Electrical and Computer Engineering University of Toronto Toronto, ON Canada; 2 School of Public Policy Simon Fraser University Vancouver, BC Canada; 3 Winterlight Labs Toronto, ON Canada; 4 Department of Psychiatry and Behavioural Neurosciences McMaster University Hamilton, ON Canada; 5 Department of Political Science Munk School of Global Affairs and Public Policy University of Toronto Toronto, ON Canada

**Keywords:** mental health, generalized anxiety disorder, impromptu speech, acoustic features, linguistic features, mobile phone

## Abstract

**Background:**

The measurement and monitoring of generalized anxiety disorder requires frequent interaction with psychiatrists or psychologists. Access to mental health professionals is often difficult because of high costs or insufficient availability. The ability to assess generalized anxiety disorder passively and at frequent intervals could be a useful complement to conventional treatment and help with relapse monitoring. Prior work suggests that higher anxiety levels are associated with features of human speech. As such, monitoring speech using personal smartphones or other wearable devices may be a means to achieve passive anxiety monitoring.

**Objective:**

This study aims to validate the association of previously suggested acoustic and linguistic features of speech with anxiety severity.

**Methods:**

A large number of participants (n=2000) were recruited and participated in a single web-based study session. Participants completed the Generalized Anxiety Disorder 7-item scale assessment and provided an impromptu speech sample in response to a modified version of the Trier Social Stress Test. Acoustic and linguistic speech features were a priori selected based on the existing speech and anxiety literature, along with related features. Associations between speech features and anxiety levels were assessed using age and personal income as covariates.

**Results:**

Word count and speaking duration were negatively correlated with anxiety scores (*r*=–0.12; *P*<.001), indicating that participants with higher anxiety scores spoke less. Several acoustic features were also significantly (*P*<.05) associated with anxiety, including the mel-frequency cepstral coefficients, linear prediction cepstral coefficients, shimmer, fundamental frequency, and first formant. In contrast to previous literature, second and third formant, jitter, and zero crossing rate for the *z* score of the power spectral density acoustic features were not significantly associated with anxiety. Linguistic features, including negative-emotion words, were also associated with anxiety (*r*=0.10; *P*<.001). In addition, some linguistic relationships were sex dependent. For example, the count of words related to power was positively associated with anxiety in women (*r*=0.07; *P*=.03), whereas it was negatively associated with anxiety in men (*r*=–0.09; *P*=.01).

**Conclusions:**

Both acoustic and linguistic speech measures are associated with anxiety scores. The amount of speech, acoustic quality of speech, and gender-specific linguistic characteristics of speech may be useful as part of a system to screen for anxiety, detect relapse, or monitor treatment.

## Introduction

### Background

Anxiety disorders are among the most common mental health issues, with an incidence of approximately 10% in the Canadian population [1]. Many Canadians are unable to access psychological and psychiatric resources to help those affected [2], in part, because of the cost of professional help [3]. It may be possible to address some of this deficit using methods that automate the measurement and diagnosis of anxiety disorders. The first step in this direction is to explore methods for the automatic detection of mental health issues that could be used to trigger early intervention, monitor treatment response, or detect relapse. In addition, frequent monitoring together with other time-series information could be used to help understand the mechanisms of generalized anxiety disorder (GAD) itself. An avenue of such automation is recording an individual’s speech and looking for signals of anxiety within the recordings.

In this work, we focused specifically on GAD [4]. A reason that GAD may be detectable in speech is that those with anxiety disorders exhibit higher activation of the sympathetic nervous system under stress than those without anxiety [5], which in turn influences the production of speech [6]. The goal of this work was to collect a large set of samples of audio speech, each with a self-reported measure of anxiety scale, and explore whether acoustic and linguistic signals correlated with measured anxiety. We built on previous studies by collecting approximately 10 times greater number of human participants than previous research on the detection of anxiety in speech. Many of the signals that we explored have been previously reported as significantly correlated with anxiety in the literature, and our goal was to leverage our larger sample size to examine which signals could be most useful in identifying anxiety in speech. We also explored linguistic indicators of anxiety that have not been considered before.

This paper is organized as follows: the next section summarizes related work in anxiety detection. The *Methods* section describes the speech sample collection methods and the set of features considered for correlation with anxiety. The *Results* section reports on the demographics of participants and feature correlations, whereas the *Discussion* section discusses the results and their implications for future research on anxiety detection. A final section provides our conclusions.

### Related Work

Although it is important to note that some scholarship is skeptical that biomarkers correlate with emotions [7], here we review existing work exploring associations between both acoustic and linguistic speech features and anxiety severity in healthy and clinical cohorts. It should be noted that these studies explore broader classes of anxiety disorders, including internalizing disorders, social phobia or social anxiety disorder (SAD), panic disorder, and agoraphobia, as well as GAD.

McGinnis et al [8] identified several acoustic characteristics of speech that can be used to detect anxiety disorders in children. Studying 71 participants aged 3 to 8 years, the researchers were able to detect internalizing disorders—a collective term for anxiety and depression—from speech. The authors extracted and selected several acoustic features from the speech produced in a 3-minute task based on the Trier Social Stress Test (TSST) for children [9]. These features included zero crossing rate, mel-frequency cepstral coefficients (MFCCs) [10], zero crossing rate for the *z* score of the power spectral density (ZCR-zPSD), dominant frequency, mean frequency, perceptual spectral centroid, spectral flatness, and the skew and kurtosis of the power spectral density. Using the Davies-Bouldin index–based feature selection [11], the MFCC features and ZCR-zPSD had the highest Davies-Bouldin score. Several models were built to predict which children had an internalizing disorder (n=43 out of 71) or were healthy. Both logistic regression and support vector machine [12] analysis achieved a classification accuracy of 80%.

Özseven et al [13] conducted a study of the speech of 43 adults aged 17 to 55 years. Of these 43 adults, 21 were clinically diagnosed with GAD, 2 were diagnosed with panic disorder, and 20 were healthy controls. The study explored 122 acoustic features derived from the participants’ speech to determine the correlation between these features and anxiety. Their results showed that 42 of the features (including MFCCs, linear prediction cepstral coefficients [LPCCs], fundamental frequency [F0], first formant [F1], second formant [F2], third formant [F3], jitter, and shimmer) showed a significant change between a neutral state and an anxious state in the participants with anxiety.

Weeks et al [14] found a relationship between anxiety and alterations in voice. Specifically, their study showed a link between vocal pitch (characterized by F0) and SAD. They collected impromptu speech samples from 46 undergraduate students, 25 with a diagnosis of SAD and 21 healthy controls. Participants also completed the Beck Anxiety Scale as a measure of self-reported anxiety severity [15]. Their results indicated that mean F0 was positively correlated (*r*=0.72; *P*=.002) with anxiety severity across all male participants. However, the correlation for female participants was weaker (*r*=0.02; *P*=.92), indicating possible sex differences in the relationship between anxiety severity and vocal pitch.

Laukka et al [16] explored the relationship between anxiety and the acoustic features of speech. They collected speech data from 71 patients with social phobia delivering public speeches and extracted 4 types of speech features: pitch (F0 mean, F0 SD, and F0 maximum), loudness (intensity mean), voice quality (HF 500, relative proportion of high-frequency spectral energy above vs below 500), and temporal aspects of speech (articulation rate and percentage of silence). The researchers observed a significant change from before treatment to after treatment (a pharmacological anxiolytic treatment for social anxiety) in F0 mean, F0 maximum, HF 500, and percentage of silence. They also calculated the Pearson correlation coefficient between state anxiety measured by the Spielberger State-Trait Anxiety Inventory [17] and the speech features. Those with a significant correlation were F0 SD (*r*=–0.24; *P*<.05) and percentage of silence (*r*=0.36; *P*<.01).

Albuquerque et al [18] investigated the relationship between acoustic speech features and anxiety. They recruited 112 adult Portuguese speakers who performed 2 tasks: reading vowels in disyllabic words and picture description. The authors extracted 18 acoustic features, including F0, F1, F2, speech duration, number of pauses, and articulation rate. They measured the percentage change between participants who were nonanxious (Hospital Anxiety and Depression Scale, Anxiety subscale [19] score ≤7) and those who were anxious (Hospital Anxiety and Depression Scale, Anxiety subscale score >7) and observed a change of >10% in speech duration.

Wörtwein et al [20] assessed the behaviors of participants experiencing anxiety caused by public speaking through audiovisual features. A total of 45 participants were recruited from Craigslist. These participants were asked to complete the Personal Report of Confidence as a Speaker scale [21], which estimates public speaking anxiety levels. Several audio features were extracted from the audio and their results showed significant relationships between the Personal Report of Confidence as a Speaker scale and SD of the 0th coefficient of the MFCC [10] (*r*=–0.36; *P*<.05), SD of F1 (*r*=–0.41; *P*<.01), and the total pause duration (*r*=0.35; *P*<.05).

Hagenaars and van Minnen [22] explored whether the activation of fear was manifested in the speech of 25 female patients diagnosed with panic disorder. Their results showed that patients with panic disorder have a significantly higher pitch (*P*<.001) during autobiographical fear memory. Respondents also spoke significantly slower (*P*<.001) during autobiographical talking than during script talking.

Di Matteo et al [23] explored the relationship between linguistic features of speech and anxiety. Their work used *passively* collected intermittent samples of audio data from participants’ smartphones, collected over a 2-week period, as input. The study had 84 nonclinical participants recruited from a web-based recruitment platform. The audio was converted to text, and the authors used the Linguistic Inquiry and Word Count (LIWC) approach [24] to classify the words into 67 different categories. They calculated correlations with 4 self-report measures: SAD, GAD, depression, and functional impairment. They observed a significant correlation between words related to perceptual processes (eg, *see* in the LIWC) with SAD (*r*=0.31; *P*=.003) and words related to rewards with GAD (*r*=–0.29; *P*=.007).

In a similar study that used LIWC features, Anderson et al [25] recruited 42 participants diagnosed with SAD and 27 healthy controls to explore the differences in the words used between these 2 groups. The participants were asked to write a distinct autobiographical and socially painful passage. They used the LIWC to extract the word count in each of the LIWC categories, such as first-person singular pronouns, anxiety-related words, and fear-related words. Their results showed that patients with SAD used more first-person singular pronouns (I, me, and mine), anxiety-related words, sensory and perceptual words, and words denoting physical touch, as well as fewer references to other people.

Overall, previous work identifies several audio features that are correlated with anxiety. However, the results are mixed because of differences in participants recruited, speech measures assessed, statistical methods used, and amount of mood induction. In addition, the largest sample size among these studies was 112, which limits the potential for generalizability to the larger population, a necessary step before considering the deployment of technologies for passive anxiety monitoring. In this study, we recruited a substantially larger cohort (n=2000) to explore features of speech from previous findings at a greater scale.

## Methods

### Data Collection

Participants from a nonclinical population were recruited for a 10- to 15-minute task implemented through a custom website. Self-report measures of anxiety were collected once at the beginning of the study and at the end of each of 2 specific tasks. In the following subsections, we describe the recruitment of participants, the data collection procedure, and the assessment of anxiety and speech measures.

### Ethics Approval

The study was approved by the University of Toronto Research Ethics Board (37584).

### Recruitment and Demographics

A total of 2000 participants were recruited using the Prolific [26] web-based human participant recruitment platform. Prolific maintains a list of registered participants and, for each participant, many characteristics, including age, income, sex, primary language spoken, country of birth, and residence. The inclusion criteria for this study were as follows: age range 18 to 65 years; fluency in English; English as a first language; and at least 10 previous studies completed on Prolific, with 95% of these previous Prolific tasks completed satisfactorily, as labeled by the study author. The data set was also balanced for sex (1000/2000, 50% female, and 1000/2000, 50% male). The Prolific platform provides us with some relevant demographics of the participants, including their age and income.

Participants who completed the study were paid £2 (US $2.74). They were able to complete the entire study remotely, using their PCs.

### Study Procedure

Participants were presented with the opportunity to participate in this study on Prolific if they met the aforementioned inclusion criteria. Those who wished to participate clicked on the study link, which brought them to a consent form that described the procedure and goals of the study and also provided information on data privacy. After they gave consent, a hyperlink brought participants to an external web application (a screenshot of which is presented in Multimedia Appendix 1) that implemented the tasks described in the following sections.

Participants were first asked to fill out the standard Generalized Anxiety Disorder 7-item scale (GAD-7) questionnaire [27], which is described in more detail in the *Anxiety Measures* section. Next, they were asked to complete 2 speech tasks, which were recorded using their computer’s internal microphone. It should be noted that our protocol also involved recording a video of the participants’ faces during both speech tasks. Although that video is not used in the work reported here, the fact that the video was requested may have influenced the set of participants willing to continue participation, as discussed later in this paper.

For the first speech task (task 1), participants were asked to read aloud a specific passage titled *My Grandfather*, which is a public domain passage that contains nearly all the phonemes of American English [28]. The full script of this passage is presented in Multimedia Appendix 2. This passage is not intended to induce stress or anxiety but to provide a baseline speech sample for each participant. It was used in this work to test the quality of the speech-to-text (STT) transcription.

For the second speech task (task 2), the participant followed a modified version of the widely used TSST [29] for the purpose of inducing a moderate amount of stress. We chose to base our anxiety stimulus on the TSST as previous studies [30,31] have shown a higher activation in participants with relatively higher anxiety after exposure to moderate stress induced by the TSST.

In this modified version of the TSST, participants were told to imagine that they were a job applicant for a job that they really want (their *dream* job) and they were invited for an interview with a hiring manager. They were given a few minutes to prepare—to decide what their *dream* job is—and how they would convince an interviewer that they are the right person for the position. Participants were also told that the recorded video would be viewed by researchers studying their behavior and language. Participants were then asked to speak for 5 minutes, making the case for themselves to be hired for that dream job.

It should be noted that in the original TSST [29], participants would normally deliver their speech in front of a live panel of judges. If a participant finished their delivery in <5 minutes, the judges in the original TSST design would encourage the participant to keep speaking for the full 5 minutes. An example statement of encouragement is as follows: “What are your personal strengths?” In our modified TSST, we implemented a similar method to encourage participants to speak for the full 5 minutes. When our software detected silence (the absence of speech for >6 seconds), it displayed several different prompts, which are reproduced in Multimedia Appendix 3, inviting participants to keep speaking on different topics related to the task. Finally, it should be noted that the modified TSST only made use of the first part of the original TSST and not the second task involving mental arithmetic.

### Anxiety Measures

Our goal was to examine possible correlations between features of speech and GAD, based largely on previously suggested features. To measure the severity of GAD, we used the GAD-7 [27], which is a 7-item questionnaire that asks participants how often they were bothered by anxiety-related problems during the previous 2 weeks. Although the 2-week period suggests that the GAD-7 measures a temporary condition, this seems to be in contradiction with the fact that a GAD diagnosis requires a 6-month duration of symptoms [32,33]. However, the GAD-7 has been validated as a diagnostic tool for GAD (using a value of 10 as the cutoff threshold) with a sensitivity of 89% and a specificity of 82% [27]. Thus, we chose to use the GAD-7 to obtain a binary label of GAD (using the same threshold of 10) as our main indicator of anxiety.

Each of the 7 questions on the GAD-7 has 4 options for the participant to select from, indicating how often they have been bothered by the 7 problems on the scale. These options and their numerical ratings are as follows: 0=not at all, 1=several days, 2=more than half the days, and 3=nearly every day. The final GAD-7 score is a summation of the values for each question, giving a severity measure for GAD in the range of 0 (no anxiety symptoms) to 21 (severe anxiety symptoms).

We also used a second, informal anxiety measure in this study to serve as an internal check to measure how much, on average, the modified TSST (task 2) induced stress and anxiety compared with task 1 (the reading or speaking of the *My* *Grandfather* passage). Here, we used a single question to measure self-reported levels of anxiety on a 4-point scale. We asked participants how anxious they felt during the task and to choose from the following numerical rating: 0=not anxious at all, 1=somewhat anxious, 2=very anxious, and 3=extremely anxious. This question was deployed immediately after the first and second tasks had been completed.

### Selection of Acoustic and Linguistic Features

#### Overview

Prior work suggested that information about the mental state of a person may be acquired from the signals within speech acoustics [34] and the language used [35]. We refer to each kind of this extracted information as a *feature* using the terminology used in the field of machine learning.

In this work, we considered both acoustic and linguistic features, which are described in the following sections. These features were extracted from each of the 5-minute speech samples in which the participant responded to the modified TSST task. It should be noted that all the participants were prompted to speak for the full 5 minutes, as described in the *Study Procedure* section, although the total speech duration of each participant may vary.

#### Acoustic Features

##### Overview

Previous research has identified several acoustic features that are correlated with anxiety, as described in the *Related Work* section. Using these previous findings as a reference point, we selected the acoustic features described in the following sections for our empirical analysis. The features were extracted using the following software packages: My-Voice Analysis [36], Surfboard [37], and Librosa [38].

##### MFCC Features

These are coefficients derived from a mel-scale cepstral representation of an audio signal. We included 13 MFCCs, a common set of acoustic signals designed to reflect changes in perceivable pitch. The MFCC features were shown to be related to anxiety in 3 studies [8,13,20]. Descriptive statistics (mean and SD) of the 13 MFCC features were used in this study. It should be noted that not all MFCC features included in this study were determined to be significant in prior work; however, these 13 are most commonly assessed together, and thus, we included them all as features of interest. The parameters we used when extracting these 13 MFCC features were as follows: window length=2048 samples, length of fast Fourier transform window=2048 samples, samples advance between successive frames=512 samples, window type=Hanning, and number of mel bands=128.

##### LPCC Features

These are coefficients derived from a linear prediction cepstral representation of an audio signal. The first 13 cepstrum coefficients were used here. The LPCC features were shown to be related with anxiety in the study by Özseven et al [13]. Descriptive statistics (mean and SD) of the 13 LPCCs were used in our study.

##### ZCR-zPSD Features

In the study by McGinnis et al [8], ZCR-zPSD was one of the top features selected using Davies-Bouldin index–based feature selection [11] for an anxiety-prediction task.

##### Amount of Speech

This refers to the amount of speech and related metrics such as the percentage of silence. These features have been shown to be related to anxiety in 3 studies [16,18,20]. Our specific feature was the amount of time, in seconds, that speech was present. We also counted the total number of words present in an STT transcript as a separate measure of the amount of speech.

##### Articulation Rate

This indicates how fast the participant spoke. The study by Hagenaars and van Minnen [22] suggested that patients with panic disorder spoke significantly slower (*P*<.001) during autobiographical talking than when reading a script.

##### F0 Feature

This is the frequency at which the glottis vibrates, also known as the *pitch* of the voice. Multiple studies have shown F0 to be one of the acoustic features affected by anxiety [13,14,16,22]. F0 varies throughout a person’s speech; therefore, both the mean and SD of F0 were used as features.

##### F1, F2, and F3 Features

These are the F1, F2, and F3 [39]. The study by Özseven et al [13] showed a significant relation with anxiety. The mean and SD of each formant were used as features.

##### Jitter

This refers to the cycle-to-cycle F0 variation of the sound wave. *Jitter* has been shown to be an indicator of anxiety [13,40,41].

##### Shimmer

This refers to the cycle-to-cycle amplitude variation of the sound wave. *Shimmer* has been shown to be related to anxiety severity [13].

##### Intensity

The squared mean of the amplitude of the sound wave within a given frame, also known as *intensity*, has been shown to be related to anxiety [16]. As the amplitude of a sound wave varies during speech, the mean and SD were used as features.

#### Linguistic Features

Using Amazon’s AWS STT [42] program, a transcript was produced from the audio recordings. From the transcripts, linguistic features were extracted using the LIWC software (Pennebaker Conglomerates, Inc) [24], which places words into dictionaries based on semantic categories. For example, 1 category is called *negemo* and contains words that relate to negative emotions, such as *hurt*, *ugly*, and *nasty*. Another category is called *health* and contains words such as *clinic*, *flu*, and *pill*. There is also a category called *anxiety,* which includes words such as *anxiety* and *fearful*. Some categories are contained within others; for example, *anxiety* is contained within *negemo*.

To apply the LIWC dictionaries, one simply counts the number of words that belong to each category, and each count becomes a feature. There are 93 categories in the LIWC, although not all are relevant for an STT transcript. We removed those features that were not relevant; for example, informal language words such as *lol* and *btw*. Other excluded categories included those related to some punctuation marks (eg, colons, quotation marks, and parentheses). After removing these, 80 linguistic features remained. Prior work [23,25], which was discussed in the *Related Work* section, has shown that LIWC categories related to perceptual processes (see, hear, and feel), words related to rewards, the use of the first-person singular pronoun, and anxiety-related words were associated with anxiety.

### Separation of Data for Analysis

The overarching objective of this study was to gain an understanding of which features of speech—both acoustic and linguistic—are correlated with the GAD-7. However, it is known that certain demographic attributes are directly indicative of anxiety. For example, sex is known to influence the prevalence of anxiety [43]. In addition, both age [44] and income [45] influence anxiety, which suggests the need to control for these demographics. An additional reason to control for the demographics is that both age and income have been shown to be related to speech features [46,47]. towing to the strong effect of sex on the GAD-7 score, we created separate data sets for analysis of female and male samples, in addition to the combined data set. We chose to do this, rather than correcting for sex computationally, because it leaves the data intact.

### Statistical Analysis

The partial Pearson correlation coefficient [48] was computed between each of the features and the GAD-7 (controlling for the effect of age and personal income). Correlations were examined for 3 versions of the data set: the entire sample data set and separately by sex for male and female participants. We considered a result statistically significant at a significance level of *P*=.05. The *P* values were not corrected to account for the large number of tests as we attempted to use features that were determined to be significant in previous works.

## Results

### Overview

This section reports the main empirical results. We begin by discussing the recruitment yield, the demographic characteristics of the participants, and the relationship between demographic attributes and the reported GAD-7 score. Next, we report correlations for the features described in the *Selection of Acoustic and Linguistic Features* section.

### Recruitment and Data Inclusion

A total of 4542 participants accepted the offer from the Prolific recruitment platform to participate in the study, of whom 2212 (48.7%) completed the study, giving a recruitment yield of approximately 49%.

Of the 2212 participants who completed the study, 2000 (90.42%) provided acceptable submissions (and thus received payment), giving a submission-to-approval yield of approximately 90%. To be clear, the recruitment continued until 2000 acceptable submissions were received. The reasons for which submissions were deemed unacceptable included the following: a missing video, a missing or grossly imperfect audio, or failure to complete one or both tasks. These acceptability criteria were distinct from those used in the subsequent review of audio quality that is described in the following paragraphs. The period of recruitment ranged from November 23, 2020, to May 28, 2021. Of note, the recruitment took place during the global COVID-19 pandemic.

In addition to the aforementioned submission approval criteria, we reviewed the input data and audio for acceptability using the following procedure. To begin, we computed all acoustic and linguistic features described in the *Selection of Acoustic and Linguistic Features* section. Recordings with poor quality were filtered out for manual review based on the following criteria:

1. A task 2 word count of <125
2. A speaking duration for task 2 of <60 seconds (compared with the full 5 minutes)
3. Any other feature value being beyond 3 SDs from the mean in either direction (outliers)

Of the 2000 participant recordings, 193 (9.65%) were flagged based on these criteria. For each of these, a researcher (BGT) listened to the task 2 audio recordings. The researcher discarded any samples that were deemed, subjectively, to be of insufficient audio quality or those whose response to task 2 was not responsive to the task itself. Of the 193 flagged participants, 123 (63.7%) were rejected through this manual review, meaning that of the 2000 samples, 1877 (93.85%) remained.

**Figure 1:**
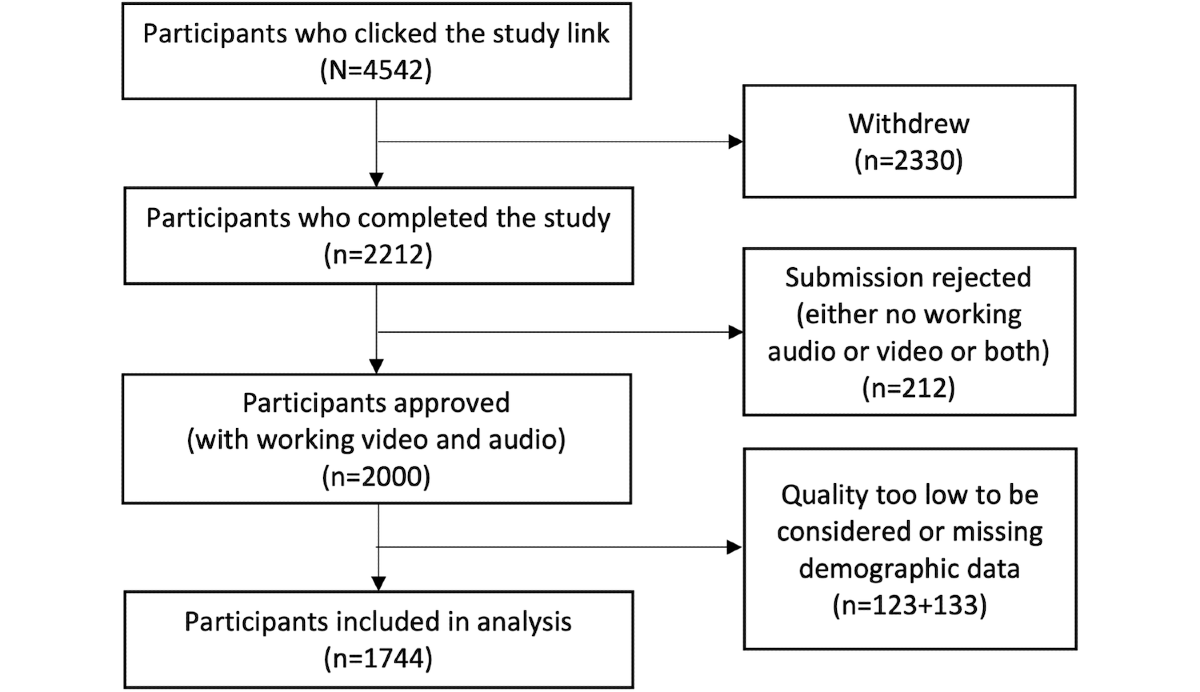
Study recruitment flow chart.

Finally, the 1877 samples were checked for missing data, and 133 (7.09%) participants had missing demographic information; consequently, the final number of participants included in our analysis was 1744 (92.91%). The flow chart of the study recruitment and quality control is presented in Figure 1. We also explored correlations of the excluded data with the GAD-7, often called missingness analysis, and this is presented in Multimedia Appendix 4.

### Data Overview and Demographics of Participants

Of the 1744 participants, 540 (30.96%) were above the GAD-7 screening threshold of 10 and 1204 (69.04%) were below the GAD-7 screening threshold of 10. Hereon, we will refer to those participants with a GAD-7 score ≥10 as the group *with anxiety* and those with a GAD-7 score <10 as the *nonanxious* group.

Table 1 shows participants’ demographics, obtained from the Prolific recruitment platform. Columns 1 and 2 of the table show the name of demographic attributes and each category, whereas columns 3 and 4 give the number (and percentage) of participants with that attribute in the group with anxiety and the nonanxious group, respectively. Column 5 gives the *P* value for a chi-square test of the null of independence to determine whether there is a significant difference between the group with anxiety and the nonanxious group for each categorical factor.

**Table 1:** Demographic characteristics of participants in the group with anxiety and the nonanxious group (N=1744).

Demographic factors		Group with anxiety (n=540), n (%)	Nonanxious group (n=1204), n (%)	*P* value from chi-square test	
**Sex**					<.001
	Male	229 (42.41)	653 (54.24)		
	Female	311 (57.59)	551 (45.76)		
**Self-reported ongoing mental health illness or condition**					<.001
	Yes	297 (55)	311 (25.83)		
	No	243 (45)	893 (74.17)		
**Personal income, pounds sterling (£1=US $1.37)**					<.001
	<10,000	181 (33.52)	281 (23.34)		
	10,000 to 19,999	112 (20.74)	208 (17.28)		
	20,000 to 29,999	92 (17.04)	259 (21.51)		
	30,000 to 39,999	60 (11.11)	184 (15.28)		
	40,000 to 49,999	36 (6.67)	109 (9.05)		
	50,000 to 59,999	20 (3.7)	74 (6.15)		
	≥60,000	39 (7.22)	89 (7.39)		
**Age (years)**					<.001
	18 to 19	27 (5)	44 (3.65)		
	20 to 29	239 (44.26)	379 (31.48)		
	30 to 39	162 (30)	334 (27.74)		
	40 to 49	67 (12.41)	219 (18.19)		
	50 to 59	39 (7.22)	132 (10.96)		
	≥60	6 (1.11)	96 (7.97)		

### Posttask Self-report Anxiety Measure

As described in the *Anxiety Measures* section, participants were asked to rate their state of anxiety after each task on a scale of 0 to 3, where 3 was the highest level of anxiety. A paired 2-tailed *t* test was conducted to assess the difference between the 2 measurements. The test validates that the modified TSST task successfully induced some anxiety in participants, with the average score on the self-reported state anxiety measure increasing from 0.5 (SD 0.6) to 1.5 (SD 0.9; *P*<.001) before and after completing task 2, respectively.

### Feature Correlations

#### Overview

The *Selection of Acoustic and Linguistic Features* section describes the set of acoustic and linguistic features that were selected. These were features that were reported as significant in prior work on anxiety and speech, as well as closely associated features. These features were computed on the speech samples of participants performing task 2—the modified TSST. The following subsections summarize the main empirical results. The correlation between demographics and the acoustic and linguistic features is presented in Multimedia Appendix 5, and the intercorrelation among the significant features is presented in Multimedia Appendix 6, Multimedia Appendix 7, and Multimedia Appendix 8 for the all-sample, female-sample, and male-sample data sets, respectively.

#### Amount of Speech

**Figure 2:**
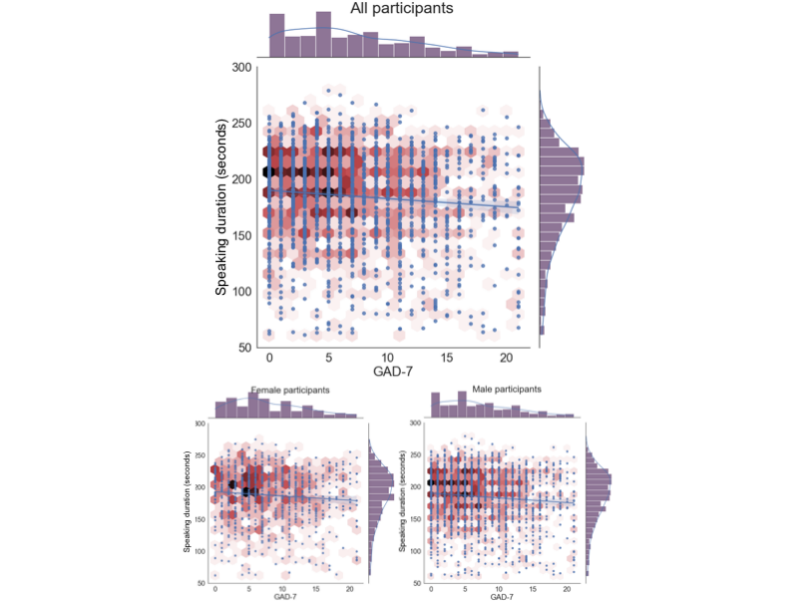
Speaking duration versus Generalized Anxiety Disorder 7-item scale (GAD-7) scatter plot and distributions.

**Figure 3:**
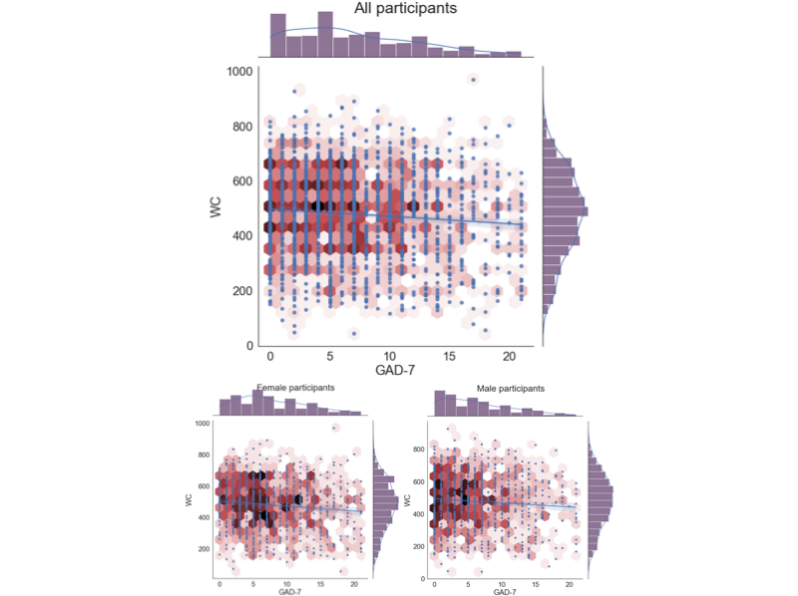
Word count (WC) versus Generalized Anxiety Disorder 7-item scale (GAD-7) scatter plot and distributions.

The features with one of the highest correlations for both the male-sample and female-sample data sets were those related to the amount the participant spoke during task 2. The 2 specific features used to estimate speech length were speaking duration (the number of seconds of speech present within the 5-minute speech task) and the word count derived from an STT transcript. Table 2 presents the correlation for the all-sample data set (controlling for sex, age, and income) and for separated female-sample and male-sample data sets (controlling for age and income). Figure 2 presents a scatter plot of speaking duration versus the GAD-7, as well as the distribution of both variables, for all 3 data sets. The scatter plot is colored to give a better sense of the density of data points. Figure 3 provides the same kind of scatter plots and distributions for the word count metric of task 2.

**Table 2:** Correlation of amount of speech features with the Generalized Anxiety Disorder 7-item scale.

Sample and feature		*r*	*P* value
**All samples (N=1744)**			
	Speaking duration	–0.12	<.001
	Word count	–0.12	<.001
**Female samples (n=862)**			
	Word count	–0.13	<.001
	Speaking duration	–0.11	<.001
**Male samples (n=882)**			
	Speaking duration	–0.13	<.001
	Word count	–0.12	<.001

#### Acoustic Feature Correlation With the GAD-7

Table 3 presents the correlation and *P* values for all the acoustic features (presented in the *Acoustic Features* section) that had *P* values above the 95% CI for the 3 data sets: all participants, female-only participants, and male-only participants. Again, it should be noted that all correlations were computed after controlling for age and personal income, whereas the calculations involving all participants also controlled for sex.

Table 4 reports results for features that previous work found to be statistically significant but for which we found no correlation in our sample. In our results, these features were not significantly associated with anxiety in any of the 3 data sets: all participants, female-only participants, and male-only participants.

Table 5 makes a direct comparison between previous work on the specific features (and their relation to anxiety) and the results from this study.

**Table 3:** Correlation of significant acoustic features with the Generalized Anxiety Disorder 7-item scale.

Sample and feature			*r*		*P* value
**All samples (N=1744)**					
	Shimmer	0.08		<.001	
	mfcc_std_2	–0.08		.002	
	mfcc_std_3	–0.07		.002	
	mfcc_mean_2	–0.07		.004	
	f0_std	0.06		.01	
	mfcc_std_5	–0.06		.01	
	mfcc_std_4	–0.05		.03	
**Female samples (n=862)**					
	mfcc_std_3	–0.10		.002	
	Shimmer	0.10		.004	
	lpcc_std_6	–0.09		.008	
	lpcc_std_4	–0.09		.008	
	mfcc_mean_2	–0.09		.01	
	Intensity_mean	–0.09		.01	
	mfcc_mean_1	–0.09		.01	
	lpcc_std_10	–0.07		.03	
	intensity_std	–0.07		.03	
	lpcc_std_12	–0.07		.04	
	mfcc_mean_8	0.07		.04	
	lpcc_mean_4	0.07		.049	
**Male samples (n=882)**					
	mfcc_std_2	–0.09		.005	
	mfcc_std_5	–0.09		.01	
	mfcc_mean_5	–0.08		.01	
	f0_std	0.07		.03	
	mfcc_std_4	–0.07		.04	
	Shimmer	0.07		.04	
	mfcc_std_11	–0.07		.046	
	f1_mean	0.07		.047	

**Table 4:** Correlation of acoustic features not found to be significant.

Feature	Previous works	This study							
		All samples			Female samples			Male samples	
		*r*	*P* value	*r*		*P* value	*r*		*P* value
Jitter	Showed a significant increase from a neutral state to an anxious state [13]	0.03	.18	–0.01		.76	0.06		.06
ZCR-zPSD^a^	ZCR-zPSD was one of the top selected features using the Davies-Bouldin index–based feature selection [8]	0.01	.67	–0.04		.29	0.05		.14
Articulation rate	Patients with panic disorder spoke significantly slower (*P*<.001) during autobiographical talking than during script talking [22]	–0.01	.64	–0.05		.12	0.02		.55
F1^b^ SD	Showed a significant change between neutral state and anxious state [13]	–0.03	.18	–0.02		.53	–0.04		.25
F2^c^ mean	Showed a significant change between neutral state and anxious state [13]	0.004	.85	0.04		.26	–0.04		.22
F2 SD	Showed a significant change between neutral state and anxious state [13]	0.01	.59	0.03		.38	–0.02		.60
F3^d^ mean	Showed a significant change between neutral state and anxious state [13]	0.02	.49	0.04		.21	–0.01		.72

^a^ZCR-zPSD: zero crossing rate for the *z* score of the power spectral density.

^b^F1: first formant.

^c^F2: second formant.

^d^F3: third formant.

**Table 5:** Comparison of previous works’ correlations with those of this study.

Feature	Previous work		This study					
			All samples		Female samples		Male samples	
	*r*	*P* value	*r*	*P* value	*r*	*P* value	*r*	*P* value
Speaking duration	–0.36	<.01	–0.12	<.001	–0.11	<.001	–0.13	<.001
MFCC^a^_std_1	–0.36	<.05	0.01	.54	0.02	.61	0.02	.52
F0^b^_mean	Female: 0.02; male: 0.72	Female: 0.92; male: 0.002	0.02	.37	–0.03	.33	0.06	.06
F0_SD	–0.24	<.05	0.06	.01	0.03	.30	0.07	.03
Intensity mean	–0.2	—^c^	–0.04	.13	–0.09	.01	0.01	.72

^a^MFCC: mel-frequency cepstral coefficient.

^b^F0: fundamental frequency.

^c^Not available.

#### Linguistic Feature Correlation With the GAD-7

The quality of the transcript produced using Amazon’s AWS STT program [42] was analyzed by comparing the transcript produced from the task 1 audio with the actual *My Grandfather* passage. The word error rate was calculated, and the STT transcript had an average word error rate of 7% (SD 4.6%).

Table 6 presents the set of linguistic features (described in the *Linguistic Features* section) that had *P* values <.05 for the same 3 data sets: all participants, male-only participants, and female-only participants. Each section in the table is sorted in decreasing order of absolute value of correlation. As described previously, the partial correlations account for age and personal income across all data sets, and we also controlled for sex in the full data set.

**Table 6:** Correlation of significant Linguistic Inquiry and Word Count linguistic features with the Generalized Anxiety Disorder 7-item scale.

Sample and feature			*r*		*P* value
**All samples (N=1744)**					
	AllPunc	0.13		<.001	
	Period	0.12		<.001	
	assent	0.10		<.001	
	negemo	0.10		<.001	
	relativ	–0.09		<.001	
	motion	–0.08		<.001	
	swear	0.08		<.001	
	anger	0.08		<.001	
	focusfuture	–0.07		.003	
	adverb	–0.07		.004	
	time	–0.07		.004	
	function	–0.07		.005	
	negate	0.07		.006	
	prep	–0.06		.007	
	WPS^a^	–0.06		.007	
	anx	0.06		.008	
	hear	0.06		.01	
	death	0.06		.01	
	ipron	–0.06		.01	
	see	–0.06		.01	
	affect	0.06		.02	
	i	0.05		.02	
	family	0.05		.02	
	sad	0.05		.03	
	ppron	0.05		.03	
	space	–0.05		.04	
	article	–0.05		.04	
	leisure	0.05		.04	
	friend	0.05		.047	
**Female samples (n=862)**					
	Period	0.16		<.001	
	AllPunc	0.14		<.001	
	adverb	–0.11		<.001	
	negemo	0.11		<.001	
	anger	0.11		.002	
	motion	–0.10		.003	
	assent	0.10		.004	
	see	–0.09		.006	
	relativ	–0.09		.006	
	sad	0.08		.01	
	Dic	–0.08		.02	
	power	0.07		.03	
	WPS	–0.07		.03	
	death	0.07		.04	
	percept	–0.07		.046	
**Male samples (n=882)**					
	AllPunc	0.13		<.001	
	assent	0.11		.001	
	relativ	–0.10		.002	
	leisure	0.10		.002	
	hear	0.10		.003	
	swear	0.10		.004	
	time	–0.10		.004	
	Apostro	0.09		.005	
	power	–0.09		.01	
	ppron	0.09		.01	
	Sixltr	–0.09		.01	
	anx	0.08		.01	
	negate	0.08		.01	
	negemo	0.08		.01	
	article	–0.08		.01	
	Period	0.08		.02	
	prep	–0.08		.02	
	focusfuture	–0.08		.02	
	family	0.08		.02	
	ipron	–0.07		.04	
	affect	0.07		.04	
	motion	–0.07		.048	

^a^WPS: words per sentence.

## Discussion

### Principal Findings

#### Overview

Our central objective was to test specific acoustic and linguistic features of impromptu speech for their association with anxiety and to do so with a larger number of participants. In this section, we discuss the implications of the findings presented in the previous section, as well as the limitations of the study.

The results presented in the *Results* section quantified the relationship between features computed from recorded speech and the self-reported GAD-7 score using Pearson correlation coefficients, controlling for age and income. The results show several significant correlations between features extracted from speech and anxiety, which can help to inform future efforts in the automatic monitoring of anxiety. We discuss these in the following sections.

#### Recruitment and Data Inclusion

Figure 1, the study recruitment flow chart, shows that the recruitment yield was 48.7% (2212/4542). Regarding the 51.3% (2330/4542) of participants who dropped out after accepting the study, we can only speculate as to why. Some may have been unwilling to have their words audio recorded or their full video recorded, and although the consent form makes this task clear, it may be that the participants who dropped out only really understood this when they saw their video on the screen.

We also conducted a missingness analysis on the 4.98% (256/4542) of samples excluded from the study (Multimedia Appendix 4). The results show that in the excluded data, the mention of words related to anxiety and those related to home had a significant positive correlation with anxiety and the count of longer words (>6 letters) was negatively correlated with anxiety. We found similar positive and negative correlations of these features in the 38.4% (1744/4542) samples included in our analysis. This indicates that excluding the 256 samples did not affect the correlation results.

#### Demographics of Participants

The proportion of participants in the group with anxiety (those above the GAD-7 screening threshold of 10) was 30.96% (540/1744), which is much higher than the general population rate of approximately 10% [1]. This result, indicating that English speakers recruited from Prolific have elevated rates of anxiety and depression, is consistent with our prior studies using recruits from Prolific and suggests that this population exhibits a higher incidence of anxiety [23,49-51]. Table 1 sheds some light on this difference: it shows that a similar high fraction of participants self-reported on their Prolific profile that they have an ongoing mental health condition.

The demographic data listed in Table 1 provide several interesting insights into the recruited cohort with respect to the presence or absence of above-threshold GAD-7 scores. First, there was a significantly larger proportion of women in the group with anxiety than men. This is consistent with previous findings suggesting that anxiety is more prevalent in women than in men [43]. We feel that this confirms that it is useful to consider separate female-only and male-only data sets to avoid the bias introduced by sex when exploring features that may correlate with the GAD-7. For example, pitch (F0) would typically be higher for women, and as a result, sex effects could easily confound the association between pitch and anxiety.

The rows in Table 1 that show the proportions of participants classified as anxious and nonanxious by income suggest that there is a relationship between income and anxiety: the 2 very lowest categories of income show a disproportionately higher amount of anxiety. There is a downward trend in anxiety with income until the very last category, which is ≥£60,000 (US $82,200). It is interesting that above a certain income level, anxiety seems to increase, although this is consistent with prior studies on anxiety and income [45].

Similarly, with respect to age, younger participants were more likely to be in the group with anxiety, which is consistent with previous work [44].

#### Posttask Self-report Anxiety Measure

As described in the *Anxiety Measures* section, we used the posttask self-reported anxiety measure as an internal check to see whether task 2 (the modified TSST task) induced more self-reported anxiety than task 1. A paired *t* test conducted on the 2 informal ratings of anxiety of the 2 tasks had a *P* value of <.001, indicating a significant difference and implying that task 2 induced greater anxiety. Recall that most of the prior work discussed in the *Related Work* section also used mood induction tasks.

#### Amount of Speech

The results suggest that features related to the amount of speech that the participants delivered in response to task 2 had one of the highest correlations with their GAD-7 response across all the features explored in this work. In particular, 2 features captured this aspect: *speaking duration* and *word count*, as shown in Table 2 (their intercorrelation with each other is presented in Multimedia Appendix 6). In all cases, the negative direction of the correlation suggests that participants who spoke more tended to have lower GAD-7 scores. This result is consistent with previous work, as shown in the first data row of Table 5; however, our study gives a much lower Pearson correlation than prior work (*r*=0.12 in this study vs *r*=0.36 in the study by Laukka et al [16]). We speculate that the more anxious a person is, the less confidence they would have about their speech; therefore, perhaps, they speak less.

#### Acoustic Features

The main purpose of this work was to explore how acoustic features relate to anxiety. We wanted to determine whether associations found in previous studies still hold with the larger sample size. Table 3 lists the features that have significant correlations, with *P*<.05, across all 3 data sets. The features with the strongest correlation in this set were *shimmer* on the all-sample data set and the SDs of the second and third MFCCs for the male-sample and female-sample data sets, respectively. We note that there are multiple parameters used in the extraction of MFCC features; therefore, a direct comparison of the specific MFCC features of our study with specific features of previous work is not possible as the prior work does not provide the exact parameters used to compute the MFCCs. The parameters used in this study are provided in the *Acoustic Features* section under the *Methods* section. That being said, in previous research, the fourth MFCC was the most significant among the 13 MFCC features in the study by Özseven et al [13] and the SD of the first MFCC in the study by Wörtwein et al [20] had a significant correlation (*r*=–0.36; *P*<.05) with an anxiety scale. These results, from both our study and previous work, suggest that signals of anxiety are present in the MFCC features.

The following features, listed as relevant in prior work, did not show significant correlations with the GAD-7: F2 and F3, jitter, ZCR-zPSD, and the articulation rate. Table 4 presents prior works’ associations with anxiety regarding these features and the correlation values obtained in our study. It is important to note that in previous research, these features were noted as significant or relevant; however, no correlations with an indicator of anxiety were provided. This makes it difficult to compare directly with the correlations obtained in our study.

#### Linguistic Features

Correlations between linguistic features extracted using the LIWC dictionaries [24] and the GAD-7 have been presented in the *Results* section. These had a higher correlation than the acoustic features, as presented in Table 6. The top LIWC category with the highest correlation in all the data sets is the count of punctuations. This includes the count of periods, which would indicate the number of separate sentences. The count of periods together with a negative correlation of words per sentence indicates that the use of shorter sentences is positively associated with anxiety.

Other LIWC categories with high correlation in the all-sample data set were negative emotion (*negemo*; eg, hurt, ugly, and nasty), anger (*anger*; eg, hate, kill, and annoyed), anxiety (*anx*; eg, worried and fearful), and sad (*sad*; eg, crying, grief, and sad). The anger, anxiety, and sad categories were constituent subsets of the negative emotion (*negemo*) category; that is, words counted under one of the anger, anxiety, or sad categories were also counted for the *negemo* category. The high intercorrelation with each other is shown in Multimedia Appendix 6. The *negemo* count had a higher correlation than these individual subcategories, suggesting that words related to anger, anxiety, and sad captured different dimensions of self-reported anxiety.

An LIWC category with a significant correlation that is present in the male-sample data set but not in the female-sample data set is the use of apostrophes (*apostro*), indicating that words with contractions (such as *I’ll*) were positively associated with the GAD-7. In addition, only for men, function words, including personal pronouns (*ppron*), had a significant positive correlation with anxiety. We speculate that male individuals with anxiety might use personal pronouns (which include I, me, and mine) to divert their attention from the anxiety-inducing event and focus on themselves. More generally, the increased use of personal pronouns has been shown to occur in individuals with depression [52], a highly comorbid mental health illness with GAD (but not only for men).

Another differentiation between men and women occurs in the LIWC feature for words related to *power* (eg, superior and bully). The *power* count had a positive correlation with the GAD-7 for women and a negative correlation for men. We speculate that the negative correlation is somehow related to the stereotypical dominance behavior associated with men.

In prior work studying associations between LIWC scores and anxiety, words related to anxiety and first-person singular pronouns were shown to be significantly associated with social anxiety [25], similar to our results. The same work has also shown that perceptual process words (see, hear, and feel) are significantly associated with anxiety, which does not align with our results. For example, the LIWC category for *see* has a negative correlation in both the all-sample and the female-sample data sets (as shown in Table 6). However, in the study by Di Matteo et al [23], the category *see* had a positive correlation (*r*=0.31; *P*=.02) with a social anxiety measure. We speculate that the use of perceptual process words (eg, *see*) might be a differentiating factor between social anxiety and GAD as it was positively correlated in the former and negatively correlated in the latter. By contrast, the LIWC category for the perceptual process word *hear* had a positive correlation in both the all-sample and the male-sample data set (also shown in Table 6). Notice that both *see* and *hear* are perceptual processes; however, the category for *see* is significant for women, whereas the category for *hear* is significant for men.

Furthermore, in prior work, death-related words were shown to have a positive correlation with anxiety [23]. Our results (as shown in Table 6) show a similar trend where death-related words had a significant positive correlation in the male-sample and all-sample data sets. However, a significant correlation was not observed in the female-sample data set.

The fact that there are several single-word categories that have significant correlations suggests that techniques that are able to look at multiple word meanings may have greater potential in making predictions.

### Limitations

A limitation of this study is the use of self-report measures to assess GAD. Self-report measures, by nature, are subjective opinions that individuals have about themselves while filling out the questionnaires and may not completely capture clinical symptoms. In this study, we took these self-report questionnaires as the true label of the audio samples. However, we believe that this is a good first step that gave us encouraging preliminary results. A psychiatric diagnosis would be an improved label but is clearly much more expensive to acquire.

A further limitation of this study is the selection bias that might be introduced during the recruitment of the participants. As presented in Figure 1, only 48.7% (2212/4542) of the participants who initially accepted the offer from Prolific to participate finished the study. We were not able to collect the GAD-7 scores of the participants who did not complete the study; therefore, we do not know their levels of anxiety. It is possible that these participants had higher levels of anxiety, which caused them to drop out of the study.

Another limitation concerns the differences in the recording devices and recording locations of the participants performing each task. Ideally, we would want every sample to be recorded using the same microphone in the same location with the same acoustics. This would reduce the potential bias introduced by different factors such as recording quality or background noise. At the same time, in a real-life scenario where an application to detect anxiety might be deployed, the recording equipment and the location will likely differ for everyone. Hence, this limitation could be unavoidable, and it might even be essential to take these types of differences into consideration.

### Conclusions

We present results from a large-N study examining the relationship between speech and GAD. Our data collection relied on participants using home recording devices, hence capturing variations in acoustic environments, which will need to be factored in when deploying tools for the detection of mental health disorders in the wild. Our goal was to provide a useful benchmark for future research by assessing the extent to which results from previous research are generalizable to our data collection approach and larger data set. We tested the most common acoustic and linguistic features associated with anxiety in previous studies and provided detailed correlation tables broken down by demographics.

Our findings are decidedly mixed. On the one hand, with our larger data set, we found modest correlations between anxiety and several features of speech, including speaking duration and acoustic features such as MFCCs, LPCCS, shimmer, F0, and F1. However, other features shown to correlate with anxiety elsewhere—including F2 and F3, jitter, and ZCR-zPSD—were not significantly associated with anxiety in our study. Although these null findings do not entirely rule out the potential of more sophisticated learning models for this task, we believe that researchers should be wary of inherent difficulties. Readers should also note that our data collection already sidestepped additional challenges that we expected to influence the detection of anxiety disorders from speech, such as variations in accents, dialects, and spoken language. On the other hand, we found statistically significant correlations for a subset of speech features from previous research. This suggests that there may be a fundamental pathway between anxiety and the production of speech, one that is robust enough to be generalized to the population.

Future investigations could explore whether features of speech from task 1 (simple reading of a passage) exhibit correlations with the GAD-7 or whether these features could be used as a control for the features of task 2 (the modified TSST task). It may also be informative to separate out different age groups (eg, younger and older) to see whether there is a specific impact of speech features on the GAD-7.

## Appendix

Multimedia Appendix 1 Web application screenshot.

Multimedia Appendix 2 My Grandfather passage.

Multimedia Appendix 3 Speech encouragement statements.

Multimedia Appendix 4 Excluded data analysis.

Multimedia Appendix 5 Correlation between demographics and acoustic and linguistic features.

Multimedia Appendix 6 Significant feature intercorrelations of the all-sample data set.

Multimedia Appendix 7 Significant feature intercorrelations of the female-sample data set.

Multimedia Appendix 8 Significant feature intercorrelations of the male-sample data set.

## Abbreviations

F0: fundamental frequency
F1: first formant
F2: second formant
F3: third formant
GAD: generalized anxiety disorder
GAD-7: Generalized Anxiety Disorder 7-item scale
LIWC: Linguistic Inquiry and Word Count
LPCC: linear prediction cepstral coefficient
MFCC: mel-frequency cepstral coefficient
SAD: social anxiety disorder
STT: speech-to-text
TSST: Trier Social Stress Test
ZCR-zPSD: zero crossing rate for the z score of the power spectral density
