# Supplementary material for: Acoustic and Linguistic Features of Impromptu Speech and Their Association With Anxiety: Validation Study
Source: JMIR Ment Health. 2022 Jul 8;9(7):e36828. doi: 10.2196/36828 (PMC9308078; doi:10.2196/36828)
Supplement: Multimedia Appendix 1 [file mental_v9i7e36828_app1.pdf]

GAD-7 Questionnaire Page

UNIVERSITY OF TORONTO | **Measuring Anxiety in Speech**

Questionaire

Please answer the questions below by selecting the response that best fits you.

**Over the last 2 weeks, how often have you been bothered by the following problems?**	Not at all	Several days	Over half the days	Nearly every day
1. Feeling nervous, anxious, or on edge	○	○	○	○
2. Not being able to stop or control worrying	○	○	○	○
3. Worrying too much about different things	○	○	○	○
4. Trouble relaxing	○	○	○	○
5. Being so restless that it's hard to sit still	○	○	○	○
6. Becoming easily annoyed or irritable	○	○	○	○
7. Feeling afraid as if something awful might happen	○	○	○	○

Next>>

Task 1 Page

UNIVERSITY OF TORONTO | **Measuring Anxiety in Speech**

For this task, read the passage below, called "My Grandfather." When you are ready to read the passage out loud, click the Record button and begin talking. Click stop after you are finished. Note that clicking Stop will submit your video recording.

My Grandfather:
You wish to know all about my grandfather. Well, he is nearly 93 years old, yet he still thinks as swiftly as ever. He dresses himself in an ancient, black frock coat, usually minus several buttons.

A long, flowing beard clings to his chin, giving those who observe him a pronounced feeling of the utmost respect. When he speaks his voice is just a bit cracked and quivers a trifle. Twice each day he plays skillfully and with zest upon a small organ.

Except in the winter when the snow or ice prevents, he slowly takes a short walk in the open air each day. We have often urged him to walk more and smoke less but he always answers, "Banana oil!" Grandfather likes to be modern in his language.

Record Stop

Task 2 Page

UNIVERSITY OF TORONTO | **Measuring Anxiety in Speech**

For this task, imagine that you are interviewing for a job you really want.

Before starting, please do the following:

1. **Decide what that "dream" job is for you.**
2. **Think about how you would convince an interviewer to hire you**

When ready, push the record button. You have 5 minutes to explain why you're the right person for the job. Be as convincing as possible. Assume that you are speaking to a person who is interviewing you for that job.

Your video will be viewed by researchers studying your behaviour and language.

Recording time countdown: 5m 0s

Record Stop

You'll have to allow your browser to use the microphone and the camera

Post-Task Self-Reported Anxiety Measure Page

UNIVERSITY OF TORONTO | **Measuring Anxiety in Speech**

Level of Anxiety

Please tell us how anxious you felt during the video recording. Anxiety is a feeling of being worried, nervous or stressed.

Not anxious at all	Somewhat anxious	Very anxious	Extremely anxious

Next>>
