# Supplementary material for: Acoustic and Linguistic Features of Impromptu Speech and Their Association With Anxiety: Validation Study
Source: JMIR Ment Health. 2022 Jul 8;9(7):e36828. doi: 10.2196/36828 (PMC9308078; doi:10.2196/36828)
Supplement: Multimedia Appendix 2 [file mental_v9i7e36828_app2.pdf]

My Grandfather:

You wish to know all about my grandfather. Well, he is nearly 93 years old, yet he still thinks as swiftly as ever. He dresses himself in an ancient, black frock coat, usually minus several buttons.

A long, flowing beard clings to his chin, giving those who observe him a pronounced feeling of the utmost respect. When he speaks his voice is just a bit cracked and quivers a trifle. Twice each day he plays skillfully and with zest upon a small organ.

Except in the winter when the snow or ice prevents, he slowly takes a short walk in the open air each day. We have often urged him to walk more and smoke less but he always answers, "Banana oil!" Grandfather likes to be modern in his language.
