# Supplementary material for: Acoustic and Linguistic Features of Impromptu Speech and Their Association With Anxiety: Validation Study
Source: JMIR Ment Health. 2022 Jul 8;9(7):e36828. doi: 10.2196/36828 (PMC9308078; doi:10.2196/36828)
Supplement: Multimedia Appendix 3 [file mental_v9i7e36828_app3.pdf]

Statements used to encourage speech when silence is detected:

- You still have some time left. Please continue!
- What are your personal strengths?
- What are your major shortcomings?
- Do you have enemies? Why?
- What do you think about teamwork?
- What do your boss/family/colleagues think about you? Why?
