# Supplementary material for: Acoustic and Linguistic Features of Impromptu Speech and Their Association With Anxiety: Validation Study
Source: JMIR Ment Health. 2022 Jul 8;9(7):e36828. doi: 10.2196/36828 (PMC9308078; doi:10.2196/36828)
Supplement: Multimedia Appendix 4 [file mental_v9i7e36828_app4.pdf]

Excluded data analysis

The following Table shows significant correlations obtained from the data that have not been included in our study based on the data inclusion steps described in Section “Recruitment and Data Inclusion.”

**N = 256**		
**Feature**	**r**	***P***
home	0.20	0.04
Sixltr	-0.19	0.04
anx	0.19	0.049
