# Supplementary material for: Acoustic and Linguistic Features of Impromptu Speech and Their Association With Anxiety: Validation Study
Source: JMIR Ment Health. 2022 Jul 8;9(7):e36828. doi: 10.2196/36828 (PMC9308078; doi:10.2196/36828)
Supplement: Multimedia Appendix 5 [file mental_v9i7e36828_app5.pdf]

Correlation between Demographics and acoustic/Linguistic features

Significant correlations (*P*<.05) are reported.

Age vs Acoustic/Linguistic features

Acoustic		
**Feature**	**r**	***P***
localabsoluteJitter	0.27	<.001
mfcc_mean_6	0.13	<.001
mfcc_std_6	-0.13	<.001
mfcc_mean_11	0.13	<.001
f0_median	-0.12	<.001
lpcc_std_3	0.12	<.001
f0_mean	-0.11	<.001
lpcc_std_2	-0.11	<.001
mfcc_mean_8	0.10	<.001
intensity_std	0.10	<.001
mfcc_std_3	-0.09	<.001
lpcc_std_10	0.09	<.001
lpcc_std_8	-0.08	0.001
f1_mean	-0.07	0.004
lpcc_mean_4	-0.07	0.005
lpcc_std_13	0.07	0.006
mfcc_mean_10	0.06	0.009
f3_stdev	-0.06	0.01
intensity_mean	0.06	0.01
mfcc_std_5	0.06	0.01
mfcc_mean_3	0.06	0.01
f2_stdev	-0.06	0.01
f0_min	-0.06	0.02
lpcc_std_9	-0.06	0.02
mfcc_std_12	0.06	0.02
lpcc_std_11	0.06	0.02
mfcc_std_11	0.05	0.02
f2_mean	-0.05	0.04
speaking_duration	-0.05	0.04
lpcc_std_5	0.05	0.04
f1_stdev	0.05	0.046
mfcc_std_8	-0.05	0.047

Linguistic		
**Feature**	**r**	***P***
compare	-0.18	<.001
insight	-0.16	<.001
conj	-0.15	<.001
see	0.14	<.001
cause	-0.14	<.001
sexual	-0.14	<.001
motion	0.13	<.001
relativ	0.13	<.001
discrep	0.13	<.001
article	0.13	<.001
they	0.12	<.001
achieve	-0.12	<.001
adverb	-0.12	<.001
focuspast	0.12	<.001
i	-0.11	<.001
work	-0.10	<.001
adj	-0.10	<.001
sad	0.10	<.001
nonflu	-0.10	<.001
number	0.10	<.001
informal	-0.10	<.001
home	0.10	<.001
social	0.10	<.001
risk	0.10	<.001
negate	0.09	<.001
verb	0.09	<.001
space	0.09	<.001
cogproc	-0.09	<.001
filler	0.09	<.001
leisure	0.08	<.001
ipron	-0.08	<.001
ingest	0.08	<.001
time	0.08	<.001
WC	-0.08	<.001
Sixltr	-0.08	0.001

percept	0.08	0.001
drives	-0.07	0.002
auxverb	0.07	0.002
quant	0.07	0.002
pronoun	-0.07	0.005
Period	0.07	0.005
you	0.06	0.006
negemo	0.06	0.006
family	0.06	0.009
power	-0.06	0.01
money	0.06	0.01
we	0.06	0.02
male	0.05	0.03
relig	0.05	0.03

Income vs Acoustic/Linguistic features

**Acoustic**		
**Feature**	**r**	***P***
f0_median	-0.22	<.001
f0_mean	-0.22	<.001
mfcc_std_5	0.20	<.001
mfcc_mean_2	0.14	<.001
localabsoluteJitter	0.13	<.001
mfcc_std_11	0.13	<.001
lpcc_std_7	0.12	<.001
speaking_duration	0.10	<.001
mfcc_std_2	0.09	<.001
mfcc_mean_1	0.08	<.001
f3_mean	-0.08	<.001
lpcc_std_3	0.08	0.001
f2_mean	-0.07	0.002
mfcc_mean_6	0.07	0.002
mfcc_std_4	0.07	0.002
f4_stdev	-0.07	0.003
mfcc_mean_4	0.07	0.004
lpcc_mean_4	-0.07	0.004
f2_stdev	-0.07	0.004
f4_mean	-0.06	0.01
f3_stdev	-0.06	0.02
f0_max	-0.06	0.02

**Linguistic**		
**Feature**	**r**	***P***
space	0.13	<.001
i	-0.13	<.001
relativ	0.13	<.001
we	0.12	<.001
WPS	0.11	<.001
negemo	-0.11	<.001
Period	-0.11	<.001
affect	-0.11	<.001
AllPunc	-0.11	<.001
article	0.11	<.001
anger	-0.10	<.001
anx	-0.10	<.001
motion	0.10	<.001
WC	0.09	<.001
ppron	-0.09	<.001
family	-0.08	<.001
female	-0.07	0.002
money	0.07	0.003
feel	-0.07	0.004
posemo	-0.07	0.005
hear	-0.06	0.007
negate	-0.06	0.009

f1_mean	-0.05	0.03
lpcc_std_12	0.05	0.03
mfcc_mean_12	0.05	0.03
lpcc_mean_5	-0.05	0.04
mfcc_mean_8	0.05	0.04

assent	-0.06	0.01
you	0.06	0.02
conj	-0.06	0.02
pronoun	-0.05	0.03
certain	-0.05	0.04
leisure	0.05	0.04
Apostro	-0.05	0.04
relig	-0.05	0.04
